# Single-molecule analysis of processive double-stranded RNA cleavage by *Drosophila* Dicer-2

**DOI:** 10.1038/s41467-021-24555-1

**Published:** 2021-07-13

**Authors:** Masahiro Naganuma, Hisashi Tadakuma, Yukihide Tomari

**Affiliations:** 1 Laboratory of RNA Function, Institute for Quantitative Biosciences, The University of Tokyo, Bunkyo-ku, Tokyo Japan; 2 School of Life Science and Technology, ShanghaiTech University, Shanghai, People’s Republic of China; 3 Department of Computational Biology and Medical Sciences, Graduate School of Frontier Sciences, The University of Tokyo, Bunkyo-ku, Tokyo Japan; 4 Present Address: RIKEN Center for Biosystems Dynamics Research, Yokohama, Japan

**Keywords:** RNA, Single-molecule biophysics

## Abstract

*Drosophila* Dicer-2 (Dcr-2) produces small interfering RNAs from long double-stranded RNAs (dsRNAs), playing an essential role in antiviral RNA interference. The dicing reaction by Dcr-2 is enhanced by Loquacious-PD (Loqs-PD), a dsRNA-binding protein that partners with Dcr-2. Previous biochemical analyses have proposed that Dcr-2 uses two distinct—processive or distributive—modes of cleavage by distinguishing the terminal structures of dsRNAs and that Loqs-PD alters the terminal dependence of Dcr-2. However, the direct evidence for this model is lacking, as the dynamic movement of Dcr-2 along dsRNAs has not been traced. Here, by utilizing single-molecule imaging, we show that the terminal structures of long dsRNAs and the presence or absence of Loqs-PD do not essentially change Dcr-2’s cleavage mode between processive and distributive, but rather simply affect the probability for Dcr-2 to undergo the cleavage reaction. Our results provide a refined model for how the dicing reaction by Dcr-2 is regulated.

## Abstract (web summary)

Fly Dicer-2 is thought to use two distinct – processive or distributive – modes of cleavage by distinguishing the terminal structures of double-stranded RNA (dsRNA) substrates with the help of its cofactor LoquaciousPD (Loqs-PD). Here the authors show by single-molecule imaging that dsRNA terminal structures and Loqs-PD change the probability for Dicer to initiate processive cleavage but not the mode of cleavage action per se.

## Introduction

The RNase III enzyme Dicer produces small interfering RNAs (siRNAs) or microRNAs (miRNAs) by cleaving long double-stranded RNAs (dsRNAs) or precursor miRNAs (pre-miRNAs), respectively^1,2^. siRNAs and miRNAs are then incorporated into Argonaute proteins to form the RNA-induced silencing complex (RISC), which recognizes complementary sequences in target mRNAs and suppresses their expression^3,4^.

Dicer enzymes are well conserved in eukaryotes, with their characteristic domain architecture comprised of an N-terminal DExD/H helicase domain, Platform domain, PAZ domain, two RNase III domains, and C-terminal dsRNA-binding domain. However, different organisms utilize Dicer in different ways. For example, humans have one Dicer, which can produce both siRNAs and miRNAs^5,6^. In contrast, *Drosophila* and other insects have two Dicer paralogs with distinct functions; Dicer-1 (Dcr-1) produces miRNAs from pre-miRNAs, whereas Dicer-2 (Dcr-2) is specialized in the processing of long dsRNAs into siRNAs^7^. Although human Dicer and fly Dcr-1 do not require ATP for cleavage^5,6,8,9^, fly Dcr-2 consumes ATP and can produce consecutive siRNAs from long dsRNAs without dissociation^10–13^. This processive cleavage reaction of Dcr-2 depends on the ATPase activity of the N-terminal helicase domain. *dcr-2* mutant flies are viable but highly susceptive to various viral infections^14,15^, highlighting the importance of the siRNA pathway in host defense against viruses.

It has been reported that the terminal structure of dsRNAs affects the cleavage reaction by Dcr-2 ^11,12,16^; in general, Dcr-2 cleaves dsRNAs with blunt ends (BLT) more efficiently than those with 2-nt 3′-overhangs (3′ovr). Previous biochemical studies have proposed that BLT dsRNAs are cleaved by Dcr-2 without dissociation (processive mode)^13^, whereas 3′ovr dsRNAs dissociate from Dcr-2 after the first cleavage and require rebinding for subsequent cleavage (distributive mode)^11,17^. This model is seemingly consistent with recent cryo-electron microscopy structures of Dcr-2 ^17^. However, direct evidence is still lacking, because the dynamic movement of Dcr-2 along dsRNAs has not been traced.

The dsRNA-binding protein Loquacious-PD (Loqs-PD) is a partner protein of *Drosophila* Dcr-2 ^18–22^, which directly binds dsRNAs and enhances the dsRNA cleavage reaction by Dcr-2 ^12,13,23,24^. Loqs-PD is required for the production of a subset of endogenous siRNAs (endo-siRNAs), especially those originating from partially self-complementary hairpin RNAs. It was proposed that Loqs-PD promotes cleavage of suboptimal dsRNA substrates by Dcr-2, altering the dependence of Dcr-2’s cleavage mode on dsRNA terminal structures^12^. However, kinetic analyses have shown that Loqs-PD decreases *K*_m_ (i.e., increases the affinity) but does not change *k*_cat_, the rate of enzyme turnover that should be influenced by different cleavage modes^13,24^. Therefore, if and how Loqs-PD converts Dcr-2’s mode of action remain unclear.

Here, we established a single-molecule imaging system for dsRNA processing by Dcr-2, which enabled us to trace substrate binding, successive cleavage, and dissociation in real-time, with precision beyond classical biochemistry. We found that the terminal structures of long dsRNAs and the presence or absence of Loqs-PD change the probability of Dcr-2 to initiate processive cleavage, but not the mode of cleavage reaction per se. Our results provide a refined and unified model of how Dcr-2 cleaves dsRNAs with different terminal structures and how Loqs-PD enhances the cleavage activity of Dcr-2.

## Results

### Fluorescent labeling does not affect the dicing reaction by Dcr-2

**Fig. 1:**
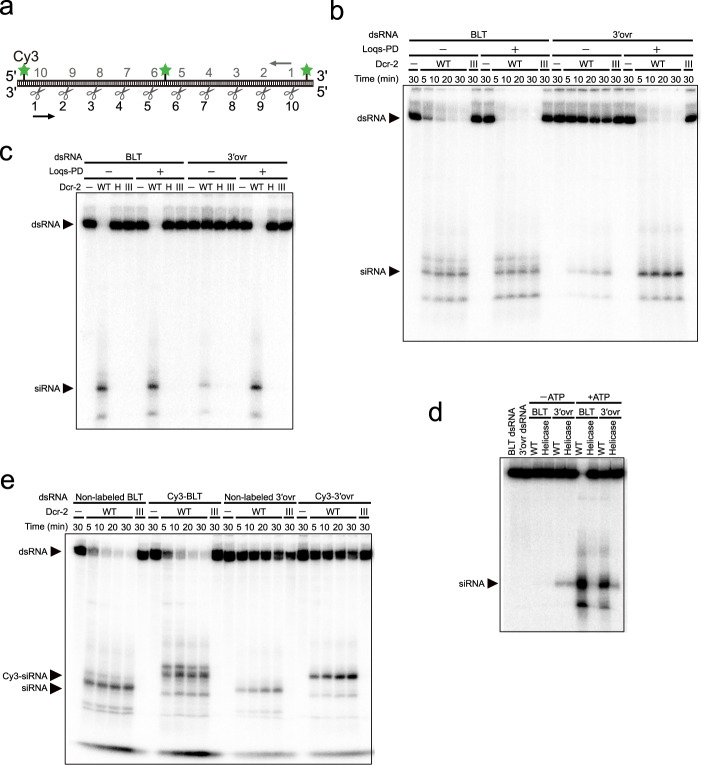
Cy3 labeling of dsRNA does not affect Dcr-2’s cleavage activity. **a** Schematic of the double-stranded RNA substrate labeled with 3× Cy3 at designated positions. Scissors represent cleavage sites. **b** dsRNA cleavage assay using wild-type Dcr-2 or the RNase III mutant (III) (~15 nM), BLT or 3′ovr dsRNAs (5 nM), and ±Loqs-PD (50 nM). *n* = 3 independent experiments. **c** dsRNA cleavage assay for the helicase (H) and RNase III mutants. Enzyme and dsRNA concentrations were the same as in **b** and the incubation time was 10 min. *n* = 2 independent experiments. **d** dsRNA cleavage assay with a higher concentration of Dcr-2, using wild-type Dcr-2 or the helicase mutant (~30 nM), and BLT or 3′ovr dsRNA (1 nM) with or without ATP. The incubation time was 120 min. *n* = 2 independent experiments. **e** dsRNA cleavage assay for the comparison between non-labeled and Cy3-labeled dsRNAs. In **b**–**e** one strand of dsRNAs was radiolabeled at the 5ʹ end and detected by phosphor imaging (see “Methods” section). *n* = 3 independent experiments. Source data are provided as a Source Data file.

To start the single-molecule analysis of the dicing reaction by Dcr-2, we sought to label Dcr-2 protein and the dsRNA substrate with two different fluorescent dyes. For this purpose, 6×His- and Halo-tagged Dcr-2 was expressed in *Drosophila* S2 cells, conjugated with biotin-Cy5 via the HaloTag ligand, and purified using Ni sepharose (Supplementary Fig. 1a, b). For the dsRNA substrate, we in vitro transcribed a 222-nt long RNA bearing Cy3 at three specific positions using Cy3-UTP and T7 RNA polymerase, and annealed it to two types of complementary strands, producing either a BLT or 3′ovr dsRNA. In theory, these symmetrical dsRNAs can be processed by 10 consecutive steps of dicing from either end; the positions of Cy3 were designed to be removed by the first or middle (sixth) cleavage by Dcr-2, or upon dissociation after the tenth cleavage, regardless of the direction of the dicing reaction (Fig. 1a). As reported previously for unlabeled Dcr-2 ^11,12^, we confirmed that biotin-Cy5-labeled wild-type Dcr-2 can cleave BLT dsRNA more efficiently than 3′ovr dsRNA (Fig. 1b) and that Loqs-PD greatly enhances the cleavage activity of wild-type Dcr-2 for 3′ovr dsRNA. In contrast, the RNase III (D1217A/D1476A) and helicase (G31R) mutants of Dcr-2 did not show detectable dicing activity in the presence or absence of Loqs-PD in our standard assay condition (~15 nM Dcr-2 and 5 nM dsRNA; 10 min) (Fig. 1c). It has been reported that the helicase mutant can distributively cleave 3′ovr dsRNAs in the absence of ATP^11,12,17^. Indeed, when we used a highly enzyme-excess condition (~30 nM Dcr-2 and 1 nM dsRNA) and a longer incubation (120 min), similar to those used in previous studies^12,17^, we were able to observe a low but detectable level of cleavage of 3′ovr dsRNA by the helicase mutant and wild-type Dcr-2 even in the absence of ATP (Fig. 1d). In the presence of ATP, cleavage of not only BLT dsRNA but also 3′ovr dsRNA by wild-type Dcr-2 was strongly enhanced (Fig. 1d), which is in contrast to some data in previous reports^12,17^ but is in fact consistent with many other previous data^11,13,16,25^ (also see “Discussion” section). Although Cy3 labeling of dsRNAs slightly enhanced the dicing activity of Dcr-2, especially for 3′ovr dsRNA, Dcr-2 still cleaved Cy3-BLT dsRNA significantly more efficiently than Cy3-3′ovr dsRNA (Fig. 1e and Supplementary Fig. 1c, d), consistent with the data using non-labeled dsRNAs (Fig. 1e and refs. ^12,17^). As previously reported^11,12^, dicing of BLT dsRNA tended to generate multiple products of various lengths in addition to the major 21–22-nt product (Fig. 1b–e), regardless of the presence or absence of the Cy3 dyes. These data indicate that fluorescent labeling of Dcr-2 and dsRNA substrates do not substantially affect the dicing activity of Dcr-2, and thus can be used for single-molecule analyses.

### Stepwise dsRNA processing by Dcr-2 at the single-molecule level

**Fig. 2:**
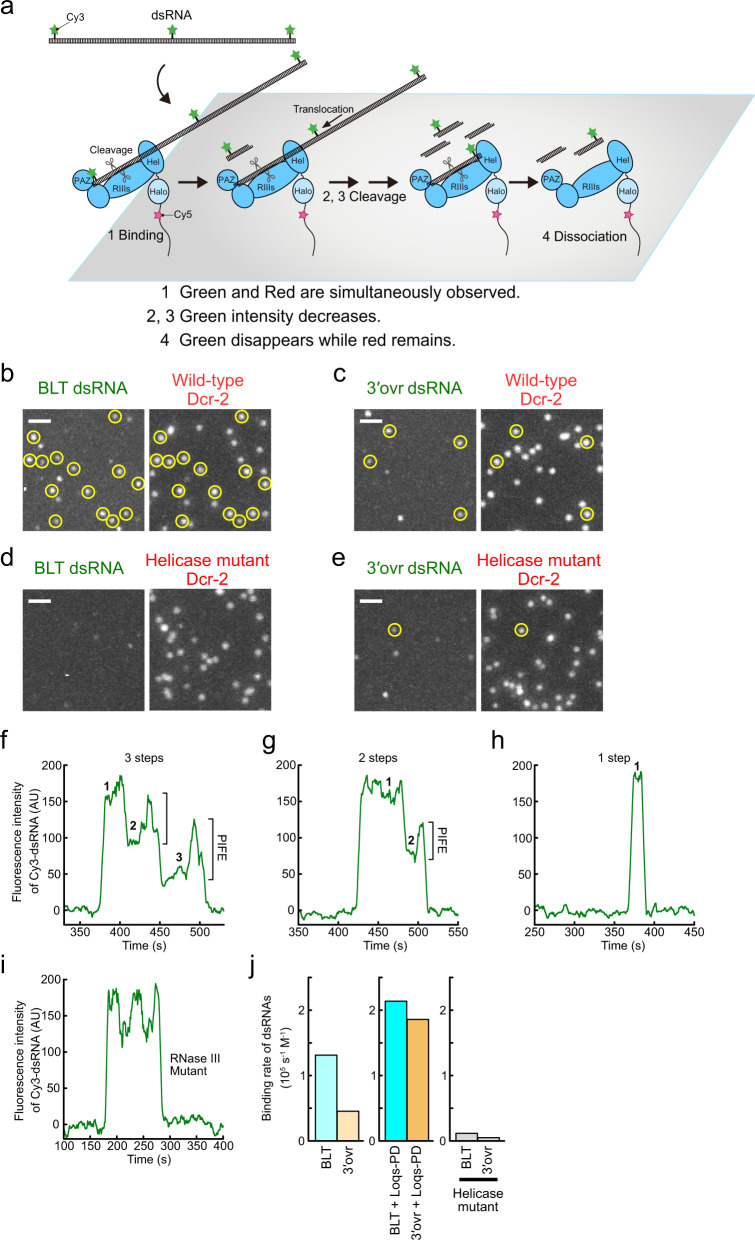
Single-molecule analysis of the dsRNA cleavage reaction by Dcr-2 tethered on the glass. **a** Schematic representation of the Dcr-2-anchored single-molecule observation. **b**–**e** Representative single-molecule images of surface-tethered Dcr-2 and co-localized dsRNAs (wild-type and BLT [**b** *n* = 9 independent experiments], wild-type and 3′ovr [**c**, *n* = 20 independent experiments], the G31R helicase mutant and BLT [**d** *n* = 2 independent experiments], and the G31R helicase mutant and 3′ovr [**e**, *n* = 2 independent experiments]). Co-localized spots were indicated by yellow circles. dsRNAs were frequently co-localized with wild-type Dcr-2, but rarely with the helicase mutant. Scale bar, 2 μm; inset. **f**–**h** Representative traces of the “3-steps” (**f**), “2-steps” (**g**), and “1-step” (**h**) events. The *x* axis shows the time after starting the observation. **i** Representative trace of 3× Cy3-labeled BLT dsRNAs co-localized with the RNase III mutant. Three repetitive PIFE events were observed without cleavage. **j** Binding rate of dsRNAs, calculated from the total number of binding events (3-steps, 2-steps, and 1-step). BLT dsRNAs bind Dcr-2 more frequently than 3′ovr dsRNAs. Loqs-PD enhances the binding frequency for both BLT and 3′ovr dsRNAs. Source data are provided as a Source Data file.

To investigate how Dcr-2 processes long dsRNAs at the single-molecule level, we utilized total internal reflection fluorescence (TIRF) microscopy. We first tethered biotin-Cy5-labeled Dcr-2 on a NeutrAvidin-derivatized quartz glass surface, added a reaction mixture containing ATP and Cy3-labeled BLT or 3′ovr dsRNAs (Fig. 2a), and monitored the reaction for 10 min. We observed many Cy3 signals of dsRNAs that co-localized with the Cy5 spots of surface-tethered wild-type Dcr-2 (Fig. 2b, c). In contrast, only few Cy3 dsRNA spots were detectable with the G31R helicase mutant of Dcr-2, suggesting that the helicase domain plays a major role in capturing dsRNA substrates (Fig. 2d, e; also see “Discussion” section).

Among the Cy3 dsRNA spots that co-localized with Dcr-2, we focused on the signals whose initial intensities corresponded to three (3×) Cy3 molecules, indicative of full-length and non-photobleached dsRNAs. As expected for progressive dicing of dsRNAs, we often observed a stepwise decrease of Cy3 dsRNA signals for wild-type Dcr-2 (Fig. 2f). Interestingly, Cy3 dsRNA signals tended to increase immediately before cleavage events by wild-type Dcr-2 (Fig. 2f), which was not observed with forced stepwise photobleaching of Cy3-dsRNAs irreversibly bound to the RNase III mutant in the presence of non-hydrolyzable ATP-γS (Supplementary Fig. 2a). Thus, the transient increase of Cy3 signals before cleavage likely reflects a physical interaction between Dcr-2 and dsRNAs, a phenomenon known as protein-induced fluorescence enhancement (PIFE)^26^. In contrast to the wild-type, the RNase III mutant of Dcr-2 in the presence of ATP showed three PIFE events without apparent decrease of the overall signals, suggesting dsRNA movement without cleavage (Fig. 2i). We concluded that our single-molecule approach is suitable for analyzing the dicing reaction by Dcr-2.

### Dcr-2 has a lower chance to initiate cleaving 3′ovr dsRNAs

**Fig. 3:**
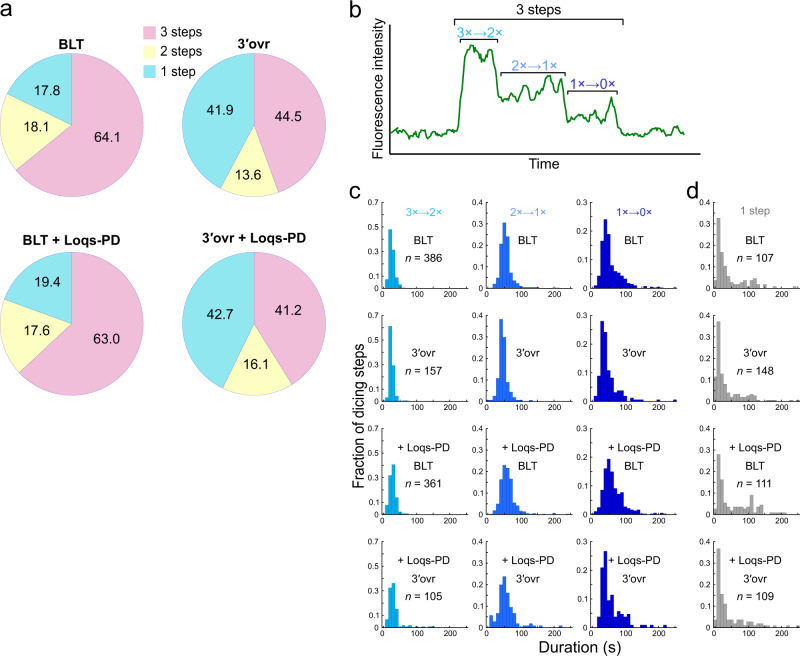
Characterization of single-molecule events with Dcr-2 tethered on the glass. **a** Pie-charts showing the proportions of the “3-steps”, “2-steps”, and “1-step” events. **b** Representative trace of the “3-steps” event. The definition of 3× → 2×, 2× → 1×, and 1× → 0× is indicated. **c** Dwell time histograms of the 3× → 2×, 2× → 1×, and 1× → 0× steps in the “3-steps” events. **d** Dwell time histograms of the “1-step” events. The dsRNA terminal structure or Loqs-PD did not change the dwell time distributions for all the steps. Source data are provided as a Source Data file.

It was previously proposed that BLT dsRNAs are processively cleaved by Dcr-2 (i.e., Dcr-2 completes consecutive dsRNA processing without dissociation), whereas 3′ovr dsRNAs are distributively processed (i.e., Dcr-2 releases the product every time it cleaves dsRNAs), based on a series of biochemical analyses on the dicing reaction^11–13^. In our single-molecule analysis, we observed three types of traces of the Cy3 dsRNA signals (Fig. 2f–h): “3-steps” in which 3× Cy3 signal appeared and decreased one by one, “2-steps” in which 3× Cy3 signal appeared, decreased to 2× or 1×, and then disappeared, and “1-step” in which 3× Cy3 signal appeared and disappeared at once. In theory, “3-steps” should indicate processive dicing of dsRNAs by Dcr-2 (note that 10 cleavage events occur during “3-steps”; Fig. 1a), while “2-steps” and “1-step” can be interpreted as distributive dicing and dsRNA dissociation without cleavage, respectively. Although these definitions can be blurred if there are extremely fast cleavage and/or dissociation events that cannot be captured by our current setting of single-molecule imaging, they appear to be quite rare (Fig. 3c and Supplementary Figs. 3 and 4; also see below).

We first simply compared the binding frequency of BLT and 3′ovr dsRNAs to Dcr-2. We picked up at least 3,000 spots of Cy5-labeled Dcr-2 in an unbiased manner, counted the events of their co-localization with Cy3 dsRNA spots, and calculated the dsRNA-binding frequency per single Dcr-2 molecule during the 10 min observation period (binding rate [*k*_on_] of dsRNA). Dcr-2 showed the binding rate of 1.3 and 0.5 × 10^5^/M/s for BLT and 3′ovr dsRNAs, respectively (Fig. 2j), suggesting that Dcr-2 prefers to bind BLT dsRNAs to 3′ovr dsRNAs. This is consistent with previous biochemical assays that showed a higher affinity of BLT dsRNAs to Dcr-2 than 3′ovr dsRNAs in the presence of ATP^12,17^.

We next analyzed the binding events of BLT and 3′ovr dsRNAs to Dcr-2 by separating them into the above-mentioned three categories. The major cleavage mode was the “3-steps” pattern for both BLT and 3′ovr dsRNAs (64.1% and 44.5%, respectively; Fig. 3a). Interestingly, we did not observe any increase in the frequency of “2-steps” events for 3′ovr dsRNAs (18.1% and 13.6% for BLT and 3′ovr dsRNAs, respectively), indicating that distributive cleavage cannot explain the lower dicing efficiency for 3′ovr dsRNAs. Instead, we found that “1-step” events occur more frequently for 3′ovr dsRNAs than for BLT dsRNAs (17.8% and 41.9% for BLT and 3′ovr dsRNAs, respectively). It was possible that this “1-step” pattern contained not only non-productive “dissociation without cleavage” events but also some productive “distributive cleavage” events, as simultaneous or immediate dissociation after the 1st cleavage may not be captured in our single-molecule observation. To clarify this point, we reversed the anchoring molecule, i.e., we now fixed dsRNA instead of Dcr-2 (Supplementary Fig. 3) so that the state of the dsRNA can be continuously monitored (e.g., after the 1st cleavage, the end-labeled fluorescent dye should disappear, decreasing the fluorescence intensity from 3× to 2×). This reversed anchoring scheme allowed us to distinguish non-productive “dissociation without cleavage” (now observed as “1-step” traces) and productive “distributive cleavage” (now observed as “2-steps” traces) (Supplementary Fig. 3e–h). Moreover, “2-steps” events can be further divided into two distinct populations: “2-steps (simultaneous)” which represents dissociation of Dcr-2 accompanied with a simultaneous single-step decrease of dsRNA intensity (Supplementary Fig. 3e; categorized as “1-step” in the original anchoring scheme), and “2-steps (delayed)” which represents a single-step decrease of dsRNA intensity followed by dissociation of Dcr-2 (Supplementary Fig. 3f, g; categorized as “2-steps” in the original scheme). Notably, we found that compared to the original anchoring scheme, the frequency of “1-step” events (dissociation without cleavage) was much lower for 3′ovr dsRNAs in the reversed scheme (Supplementary Fig. 4a), presumably due to different molecular environments on the glass surface, including the non-specific affinity of dsRNA or Dcr-2 to the surface. Nevertheless, as observed in the original scheme, the frequency of “2-steps” events was similarly rare between BLT and 3′ovr dsRNAs in the reversed scheme, while processive cleavage (“3-steps” events) was still the major mode for both types of dsRNAs. We concluded that Dcr-2 simply has a lower probability to start cleaving 3′ovr dsRNAs compared to BLT dsRNAs, rather than that Dcr-2 prefers to cleave 3′ovr dsRNAs in a distributive manner.

### dsRNA terminal structures do not change the property of processive cleavage

Our data have revealed that Dcr-2 can processively cleave not only BLT dsRNAs but also 3′ovr dsRNAs. To know if the speed and other properties of processive cleavage are different between BLT and 3′ovr dsRNAs, we focused on the “3-steps” events and analyzed the dwell time for each step, i.e., 3× → 2×, 2× → 1×, and 1× → 0× of the Cy3 dsRNA signals (Fig. 3b, c). At any of these three steps, the peak positions of the dwell time distributions were virtually the same between BLT and 3′ovr dsRNAs (Fig. 3c). Similar dwell time distributions were observed in the reversed anchoring scheme where dsRNAs were fixed instead of Dcr-2 (Supplementary Fig. 4b, c). Based on the data from both the original and reversed anchoring schemes, we estimated that it takes ~10 s for Dcr-2 to make one cleavage (i.e., to produce each siRNA) in our current single-molecule observation setting. When we lowered the ATP concentration from 1 mM to 10 μM (note that the reported *K*_m_ value for ATP is 14 μM^13^), the speed of cleavage was markedly slowed down, as is expected for ATP-driven processive cleavage (Supplementary Fig. 6; please note that the binding frequency is also dramatically reduced by low ATP, as anticipated for ATP-dependent helicases, so only a limited number of traces could be analyzed).

Finally, we utilized the Förster resonance energy transfer (FRET) to validate the dwell time in the processive cleavage reaction. In the reversed anchoring scheme, Alexa 660-labeled Dcr-2 translocates on Cy3-labeled dsRNA, producing the FRET signal between the two dyes when they become close to each other along with the movement of Dcr-2. As expected, we observed three FRET peaks during the cleavage process, and the dwell time between each peak estimated from FRET signals matched well to that estimated by PIFE signals (Supplementary Fig. 5). Taken all together, we concluded that, once Dcr-2 initiates processive cleavage, the property of the dicing reaction is not influenced by the initial terminal structure of dsRNAs.

We next focused on the dwell time of “1-step” events, which mostly represent binding and dissociation between Dcr-2 and dsRNAs without cleavage. Again, the peak dwell time was essentially the same between BLT and 3′ovr dsRNAs in the original scheme (Fig. 3d; Dcr-2 anchoring) and the reversed scheme (Supplementary Fig. 4c; dsRNA anchoring), indicating that the property of non-productive binding is also independent of dsRNA terminal structures.

### Loqs-PD simply increases the dsRNA-binding frequency of Dcr-2

It was previously shown that Loqs-PD enhances the cleavage activity of Dcr-2 ^13,16,23,24^, especially for 3′ovr dsRNA substrates^12^. To know how Loqs-PD changes Dcr-2’s molecular behavior, we performed single-molecule analysis of the dicing reaction in the presence of Loqs-PD. Consistent with previous biochemical studies^12,13,24^, Loqs-PD increased the overall binding frequency of Dcr-2 (Fig. 2j). This effect was more pronounced for 3′ovr dsRNAs (~3.7 fold) than BLT dsRNAs (~1.7 fold). However, the proportions of “1 step”, “2 steps”, and “3 steps” events remained essentially unchanged in the presence or absence of Loqs-PD, for both 3′ovr and BLT dsRNAs, in the original and reverse anchoring schemes (Fig. 3a and Supplementary Fig. 4a). Moreover, the peak dwell times were largely unaffected by Loq-PD, for each step in processive cleavage (Fig. 3c and Supplementary Fig. 4b) as well as for non-productive binding (Fig. 3d). These data suggest that, although Loqs-PD increases the initial dsRNA-binding rates of Dcr-2, it does not change its processivity, regardless of the dsRNA terminal structures.

## Discussion

**Fig. 4:**
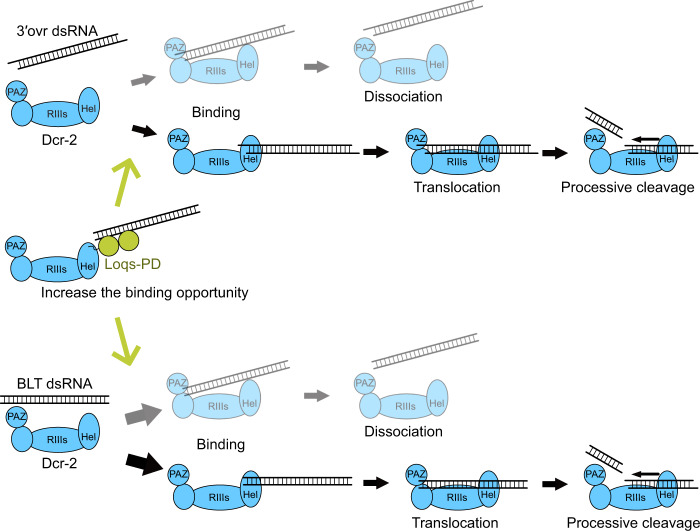
A model for modulation of Dcr-2’s cleavage activity by the dsRNA terminal structures and Loqs-PD. BLT dsRNAs have a higher chance to be cleaved than 3′ovr dsRNAs. A common mechanism of processive cleavage is used for both BLT and 3′ovr dsRNAs. Loqs-PD simply increases the binding opportunity for both BLT and 3′ovr dsRNAs.

It has been thought that Dcr-2 cleaves dsRNAs in two distinct modes, depending on the terminal structures: the processive mode for BLT dsRNAs and the distributive mode for 3′ovr dsRNAs. This conclusion was based on bulk biochemical experiments, in which Dcr-2 was allowed to cleave radiolabeled dsRNAs for a short time and then challenged by a vast excess of cold dsRNAs. However, such a pulse-chase assay cannot formally distinguish whether the dissociation of Dcr-2 from radiolabeled dsRNAs occurred before or after the initiation of the cleavage reaction. Our single-molecule analysis revealed that, although the overall binding frequency of 3′ovr dsRNAs is ~3-fold lower than BLT dsRNAs (Fig. 2j), 3′ovr dsRNAs can be processively cleaved by Dcr-2 just like BLT dsRNAs. Moreover, Loqs-PD did not switch Dcr-2’s mode of action but merely increased the overall binding frequency of dsRNAs to Dcr-2 (Figs. 2j and 3a). Indeed, the relative probability of processive cleavage as well as the dwell time of each step in the cleavage reaction was essentially unaffected by different dsRNA terminal structures or Loqs-PD (Fig. 3c). These results suggest that, once Dcr-2 initiates a processive cleavage reaction, a common molecular mechanism is used to complete the reaction. In other words, the difference between BLT and 3′ovr dsRNAs and the modulation by Loqs-PD can simply be attributed to the probability for Dcr-2 to undergo the cleavage reaction (Fig. 4).

Structural analysis has indicated that to achieve processive dicing, the terminal of BLT dsRNAs is first captured by the helicase domain of Dcr-2 and then threaded through the helicase domain to the Platform-PAZ domains^17^. Our single-molecule imaging data suggest that not only BLT dsRNAs but also 3′ovr dsRNAs can undergo this same process with the same dwell time, albeit less frequently (Figs. 2j and 3a, c). Interestingly, we observed repetitive PIFE events without a decrease in the overall signals for the RNase III mutant of Dcr-2 (Fig. 2i), suggesting that continuous dsRNA threading through the helicase domain occurs even without actual cleavage.

The distributive mode of cleavage, which does not require ATP or the helicase activity of Dcr-2, is thought to occur when the terminal of 3′ovr dsRNAs is first captured by the Platform-PAZ domains. In some previous studies, distributive cleavage was reported to be the major mode for 3′ovr dsRNAs^12,17^. However, in our hands, distributive cleavage was detectable but rare for both 3′ovr and BLT dsRNAs (Figs. 1d, 3a and Supplementary Fig. 4a). It was reported that pre-*let-7* bearing a short double-stranded stem region of ~22-nt and a 3′ovr dsRNA of 30-nt are cleaved by Dcr-2 in an ATP-independent manner but long dsRNAs of 106 nt in size require ATP hydrolysis for processing^13,25^, suggesting that the substrate length determines the major mode of cleavage by Dcr-2. In our current biochemical and single-molecule analyses, we used 220-nt dsRNAs, which are relatively long among those used in previous studies. Notably, replication of flock house virus produces a ∼400 bp dsRNA, which serves as the major Dcr-2 substrate in infected *Drosophila*^27^. Given that Dcr-2 plays a critical role in antiviral immunity in insects^14,15^, ATP-dependent processive cleavage as the major mode of reaction would be suitable for Dcr-2 to process virus-derived long dsRNAs regardless of their terminal structures.

Loqs-PD increased the overall binding frequency (Fig. 2j), but did not change the ratio of processive cleavage and non-productive binding events (Fig. 3a), for both BLT and 3′ovr dsRNAs. Given that Loqs-PD alone can bind dsRNAs^23^, we envision that Loqs-PD brings dsRNAs close to Dcr-2, increasing the opportunity for Dcr-2 to bind dsRNAs either via the helicase domain or the Platform-PAZ domains (Fig. 4). In summary, our single-molecule analysis provides a refined, simple model for how Dcr-2’s cleavage reaction is modulated by dsRNA terminal structures and Loqs-PD.

## Methods

### Plasmid constructions

pCold-HisHRV-3C-Loqs-PD was a kind gift from Dr. Fukunaga^13^. The pASHaloW Gateway destination vector was generated by inserting a DNA fragment containing the Halo tag and TEV protease recognition sequence from pFN18A (Promega) into pASW^28^, at a 3′ downstream site of the SBP sequence by In-FusionHD (Clontech). To construct pASHaloW-Dcr-2, a DNA fragment containing the Dcr-2 gene was amplified from pASW-Dcr-2^29^ and cloned into pENTR/D-TOPO (Invitrogen), followed by recombination with pASHaloW with Gateway LR Clonase II (Invitrogen). pAHisHaloW-Dcr-2 was constructed from pASHaloW-Dcr-2 by substituting the SBP sequence with 6×His. The G31R (helicase) and D1217A/D1476A (RNase III) mutations were introduced by QuikChange site-directed mutagenesis (Stratagene) (Supplementary Table 1).

### Western blot analysis

Anti-Dcr-2^30^ (1:500) and anti-Loqs^30^ (1:1000) antibodies were used as primary antibodies. Anti-IgG (H + L chain) (Mouse) pAb-HRP (MBL) (1:1000) was used as a secondary antibody. Signal Enhancer HIKARI (Nacalai tesque) was used for Dcr-2 detection. Chemiluminescence was induced by Luminata Forte Western HRP Substrate (Millipore), and images were acquired by Amersham Imager 600 (GE healthcare).

### Protein labeling with Halo-tag ligands

For single-molecule analysis, lysate from S2 cells, in which pAHisHaloW-Dcr-2 wild-type, G31R, or D1217A/D1476A was overexpressed, was incubated with 0.5 µM HaloTag Cy5-biotin ligand^31^ for Dcr-2-tethered experiments, or 35 µM HaloTag Alexa fluor 660 ligand (Promega) for dsRNA-tethered experiments, at 25 °C for 30 min. The labeled proteins were subjected to SDS-PAGE and visualized by a LAS-3000 image system (Fujifilm). Free ligands were removed by purification using cOmplete His-Tag Purification Resin (Sigma-Aldrich), and the labeled proteins were supplemented with 10% glycerol, 0.2 mg/ml BSA, shock-frozen, and stored at −80 °C.

### dsRNA preparation

For Dcr-2-anchored single-molecule experiments, a 222-nt template DNA was designed and cloned into pUC57 (GenScript): GGGCCTCAAGACAGCGAGACCGCGAGAGAGCGAGCCGCACGCCACCAACGGCAACGCACAGAGCCCACGCAGGACAACGACGGGAAGAAAGACCGGCAGCAGCCGGAATGCACGCCACACGACCGAAGAGCCAACGGACCAGCAGCCAGCAACAGCACAGACCGAGCGCAACGAACCAGGAGACCGCGAAGCAGCAGCACCACCACCGGCAACGTCAAGCCC. The DNA primers (Supplementary Table 1), GTACTTAATACGACTCACTATAGGGCCTCAAGACAGCGAG, and G(2′-*O*-Me-G)GCTTGACGTTGCCGG, were used for amplifying the template DNA for the forward RNA strand (to be Cy3-labeled). The DNA primers, GTACTTAATACGACTCACTATAGGGCTTGACGTTGCCGGT and G(2′-*O*-Me-G)GCCTCAAGACAGCGAG, were used for the reverse strand of BLT dsRNA, and GTACTTAATACGACTCACTATAGCTTGACGTTGCCGGTGG and T(2′-*O*-Me-T)GGGCCTCAAGACAGCGAG for that of 3′ovr dsRNA. For dsRNA-anchored single-molecule experiments, a 222-nt template DNA was designed and cloned into pUC57 (GenScript): GGGCCACAAGACAGCGAGACCGCGAGAGTGCGAGCCGCACGCCACCAACGGCAACGCACAGAGCCCACGCAGGACAACGACGGGAAGAAAGACCGGCAGCAGCCGGAATGCACGCCACACGACCGAAGAGCCAACGGACCAGCAGCCAGCAACAGCACAGACCGAGCGCAACGAACCAGGAGACCGCGAAGCAGCAGCACCACCACCGGCAACGTCAAGCCC. The DNA primers, GTACTTAATACGACTCACTATAGGGCCACAAGACAGCGAG and G(2′-*O*-Me-G)GCTTGACGTTGCCGG, were used for amplifying the template DNA for the forward RNA strand (to be Cy3-labeled). The DNA primers, GTACTTAATACGACTCACTATAGGGCTTGACGTTGCCGGT and G(2′-*O*-Me-G)GCCACAAGACAGCGAG, were used for the reverse strand of BLT dsRNA, and GTACTTAATACGACTCACTATAGCTTGACGTTGCCGGTGG and T(2′-*O*-Me-T)GGGCCACAAGACAGCGAG for that of 3′ovr dsRNA. RNAs were in vitro transcribed by T7-Scribe Standard RNA IVT Kit (CELLSCRIPT) in the presence of 15 mM GMP to produce 5′ monophosphorylated RNAs. For the transcription of 3× Cy3-labeled RNAs, UTP was omitted, and instead 1.5 mM UTP-Cy3 (Amersham) was added. The labeling efficiency was estimated as nearly 100%, based on the absorbance at 260 nm and 550 nm. To prepare dsRNAs, pairs of ssRNAs were annealed by heating at 95 °C for 5 min in lysis buffer (30 mM HEPES-KOH (pH 7.4), 2 mM MgOAc, 100 mM KOAc) and cooling slowly. For Fig. 1b–e, the forward RNA strands were 5′-^32^P radiolabeled using [γ-^32^P]ATP (6000 Ci/mmol; PerkinElmer) and T4 polynucleotide kinase (TAKARA) by the 5′ phosphate exchange reaction and gel-purified. For dsRNA-anchored experiments, dsRNA was labeled with (+)-Biotinamidohexanoic acid hydrazide (Sigma) by the 3′ end-labeling method reported previously^32^.

### dsRNA cleavage assay

The dicing reaction mixture contained 1 mM TCEP, 12 mM MgOAc, 1 mg/ml BSA, 100 mM KOAc, 30 mM HEPES-KOH (pH 7.4), 0.1 U/μl RNasin (Promega), 25 mM creatine phosphate (Sigma), 0.03 U/μg creatine kinase, 13.5–22 nM Dcr-2, 1 mM ATP, and 5 nM radiolabeled dsRNA. For the +Loqs-PD conditions, a 2.5-fold excess amount of Loqs-PD was mixed with Dcr-2 and incubated for 10 min on ice before initiating the cleavage reaction by adding the dsRNA substrate. At each time point, 5 μl aliquots were taken from the reaction mixture and quenched by the addition of 5 μl of 2× low salt PK solution (0.2% SDS, 20 mM EDTA, 20 mM HEPES-KOH (pH7.4), 20% Proteinase K), and immediately incubated at 55 °C for 10 min. The samples were mixed with 10 µl of 2× formamide dye (10 mM EDTA (pH 8.0), 98% w/v deionized formamide, 0.025% w/v xylene cyanol, 0.025% bromophenol blue), incubated at 68 °C for 5 min, and analyzed by denaturing PAGE.

### General procedures for single-molecule image acquisition

Single-molecule images were visualized by a total internal reflection fluorescence microscope equipped on an inverted type microscope (IX71, Olympus) with a ×150 oil immersion objective lens (UAPON 150×OTIRFM, NA 1.45, Olympus), as previously described^29^, except that additional MS(PEG)4 (Thermo, 333 Da) treatment was performed right before using a slide for dsRNA fixing experiments. The Cy3 and Cy5/Alexa 660 dyes were illuminated simultaneously with an DPSS laser (515 nm; Fandango150, Cobolt) and a helium–neon (He–Ne) laser (633 nm; GLG5410, SOC), respectively. Fluorescence images from Cy3 and Cy5/Alexa 660 were separated by using a DualView2 (Optical Insights) and then projected side-by-side onto a back-illuminated electron-multiplying charge-coupled device (EMCCD) camera (iXon3 DU-897E-CSO-#BV, 512 × 512 pixels, Andor Technology).

### General data analysis procedures for single-molecule data

Images were analyzed using ImageJ software (http://rsb.info.nih.gov/ij/) with a built-in function and custom-designed plug-in software. The fluorescent intensity of the spots was measured using 6-pixels diameter circular regions of interest (ROIs). Only the spots with fluorescence intensities equivalent to 3× Cy3 (evaluated from “3-steps” cleavage events) were analyzed using Microsoft Excel 2016. All graphs were generated using KaleidaGraph (Synergy Software).

### Measuring the steps and time constants of photobleaching for Cy3-dsRNAs

The observation mixture (0.03 U/ml creatine kinase, 0.1 U/ml RNasin Plus, 10 mM MgOAc. 1% Biolipidure-203 (NOF Corporation), 1 mM TCEP, 5 mM protocatechuic acid, 50 nM protocatechuate-3,4-dioxygenase, 5 mM Trolox in lysis buffer) was pre-mixed at 25 °C. Then, 2–3 nM Cy5-biotin-labeled Dcr-2 D1217A/D1476A was infused into the observation chamber. After the observation chamber was washed twice with lysis buffer containing 1 mM TCEP and rinsed with the observation mixture, the observation mixture containing 2 nM Cy3-labeled BLT dsRNA and 1 mM ATP-γS, was infused into the chamber. Then images were taken continuously for 1200 s at a frame rate of 2 frame/s with the DPSS laser at three different power (16, 24, and 32 mW) and the He–Ne laser at 0.8 mW. Only the first and second photobleaching times were measured, because the third photobleaching and dissociation could not be discriminated. The mean fluorescence in each image was plotted versus time, and the resulting curve yielded a time constant of photobleaching in each condition. The inverse of photobleaching time constants for Cy3 versus the power was plotted (Supplementary Fig. 2b).

### Continuous monitoring of spot appearance

For Dcr-2 fixing experiments, 500–900 nM Cy5 biotin-labeled Dcr-2 was diluted 300-fold with lysis buffer immediately before immobilization. After the observation chamber was washed twice with lysis buffer containing 1 mM TCEP and rinsed with the observation mixture, the observation mixture containing 2 nM Cy3-labeled BLT or 3ʹovr dsRNA and 1 mM ATP was infused into the chamber. We made sure that no signal of Cy5 is observed in the experiments without Cy5 biotin-labeled Dcr-2 or NeutrAvidine (Supplementary Fig. 2c). Images were continuously taken for 600 s at a frame rate of 1 frames/s at a power of 4 mW for the DPSS laser and 0.8 mW for the He–Ne laser. Stage drift was corrected using a slice alignment plugin^33^. To analyze single-molecule association, dissociation, and cleavage events, an average intensity projection of the first 50 frames in stack was first created. Then, the Cy5 fluorescent spots were automatically picked up by using a custom-made macro. The Cy3 spots that displayed co-localization with Cy5 within the entire view field (512 × 256 pixels; 1450 μm^2^) were analyzed. Then, the integrated intensity traces were generated to identify cleavage events of Cy3-labeled dsRNAs. By referencing the idealized traces generated by vbFRET^34^, a hidden Markov model-based analysis package on MATLAB, the duration times of 3× → 2× (t2–t1), 2× → 1× (t3–t2), and 1× → 0× (t4–t3) were determined by finding the appearance (t1), first decrease (t2), second decrease (t3), and disappearance (t4) times of the Cy3 signals. dsRNA-anchoring single-molecule imaging was performed basically in the same method as Dcr-2-anchoring single-molecule imaging except for the following modification. 200 nM Cy3 biotin-labeled dsRNA was diluted 400-fold with lysis buffer immediately before immobilization. After the observation chamber was washed, the observation mixture containing ~25 nM Alexa 660-labeled Dcr-2 and 1 mM ATP was infused into the chamber. Images were continuously taken for 1200 s at a frame rate of 0.5 frames/s at a power of 2 mW for the DPSS laser and 0.4 mW for the He–Ne laser. For data analysis of dsRNA anchoring, the duration times of Cy3-PIFE and Alexa 660-FRET signal were determined using the raw traces.

### Reporting summary

Further information on research design is available in the Nature Research Reporting Summary linked to this article.

## Supplementary information

Supplementary Figures 1-6 and Supplementary Table 1

Reporting Summary

## Data Availability

The data supporting the findings of this study are available from the corresponding authors upon reasonable request.

## Code availability

For single-molecule analysis, regions of interest (ROIs) were drawn using ImageJ, and the intensity traces were generated to identify the cleavage reaction. Macro codes are available from the corresponding authors upon reasonable request. vbFRET, a hidden Markov model-based analysis software, which was used to generate idealized traces for reference is available from the website (http://vbfret.sourceforge.net/).

## References

[1] Ha M, Kim VN (2014). Regulation of microRNA biogenesis. Nat. Rev. Mol. Cell Biol..

[2] Wilson RC, Doudna JA (2013). Molecular mechanisms of RNA interference. Annu. Rev. Biophys..

[3] Carthew RW, Sontheimer EJ (2009). Origins and mechanisms of miRNAs and siRNAs. Cell.

[4] Kawamata T, Tomari Y (2010). Making RISC. Trends Biochem. Sci..

[5] Zhang H, Kolb FA, Brondani V, Billy E, Filipowicz W (2002). Human Dicer preferentially cleaves dsRNAs at their termini without a requirement for ATP. EMBO J..

[6] Provost P (2002). Ribonuclease activity and RNA binding of recombinant human Dicer. EMBO J..

[7] Lee YS (2004). Distinct roles for Drosophila Dicer-1 and Dicer-2 in the siRNA/miRNA silencing pathways. Cell.

[8] Jiang F (2005). Dicer-1 and R3D1-L catalyze microRNA maturation in Drosophila. Genes Dev..

[9] Tsutsumi A, Kawamata T, Izumi N, Seitz H, Tomari Y (2010). Recognition of the pre-miRNA structure by Drosophila-Dicer-1. Nat. Struct. Mol. Biol..

[10] Liu Q (2003). R2D2, a bridge between the initiation and effector steps of the Drosophila RNAi pathway. Science.

[11] Welker NC (2011). Dicer’s helicase domain discriminates dsRNA termini to promote an altered reaction mode. Mol. Cell.

[12] Sinha NK, Trettin KD, Aruscavage PJ, Bass BL (2015). Drosophila dicer-2 cleavage is mediated by helicase- and dsRNA termini-dependent states that are modulated by loquacious-PD. Mol. Cell.

[13] Cenik ES (2011). Phosphate and R2D2 restrict the substrate specificity of Dicer-2, an ATP-driven ribonuclease. Mol. Cell.

[14] Wang XH (2006). RNA interference directs innate immunity against viruses in adult Drosophila. Science.

[15] Galiana-Arnoux D, Dostert C, Schneemann A, Hoffmann JA, Imler JL (2006). Essential function in vivo for Dicer-2 in host defense against RNA viruses in Drosophila. Nat. Immunol..

[16] Fukunaga R (2018). Loquacious-PD removes phosphate inhibition of Dicer-2 processing of hairpin RNAs into siRNAs. Biochem. Biophys. Res. Commun..

[17] Sinha NK, Iwasa J, Shen PS, Bass BL (2018). Dicer uses distinct modules for recognizing dsRNA termini. Science.

[18] Hartig JV, Esslinger S, Böttcher R, Saito K, Förstemann K (2009). Endo-siRNAs depend on a new isoform of loquacious and target artificially introduced, high-copy sequences. EMBO J..

[19] Zhou R (2009). Processing of Drosophila endo-siRNAs depends on a specific Loquacious isoform. RNA.

[20] Hartig JV, Förstemann K (2011). Loqs-PD and R2D2 define independent pathways for RISC generation in Drosophila. Nucleic Acids Res..

[21] Okamura K (2008). The Drosophila hairpin RNA pathway generates endogenous short interfering RNAs. Nature.

[22] Miyoshi K, Miyoshi T, Hartig JV, Siomi H, Siomi MC (2010). Molecular mechanisms that funnel RNA precursors into endogenous small-interfering RNA and microRNA biogenesis pathways in Drosophila. RNA.

[23] Trettin KD, Sinha NK, Eckert DM, Apple SE, Bass BL (2017). Loquacious-PD facilitates Drosophila Dicer-2 cleavage through interactions with the helicase domain and dsRNA. Proc. Natl Acad. Sci. USA.

[24] Fukunaga R (2012). Dicer partner proteins tune the length of mature miRNAs in flies and mammals. Cell.

[25] Fukunaga R, Colpan C, Han BW, Zamore PD (2014). Inorganic phosphate blocks binding of pre-miRNA to Dicer-2 via its PAZ domain. EMBO J..

[26] Hwang H, Myong S (2014). Protein induced fluorescence enhancement (PIFE) for probing protein-nucleic acid interactions. Chem. Soc. Rev..

[27] Aliyari R (2008). Mechanism of induction and suppression of antiviral immunity directed by virus-derived small RNAs in Drosophila. Cell Host Microbe.

[28] Iwasaki S (2010). Hsc70/Hsp90 chaperone machinery mediates ATP-dependent RISC loading of small RNA duplexes. Mol. Cell.

[29] Iwasaki S (2015). Defining fundamental steps in the assembly of the Drosophila RNAi enzyme complex. Nature.

[30] Miyoshi K, Okada TN, Siomi H, Siomi MC (2009). Characterization of the miRNA-RISC loading complex and miRNA-RISC formed in the Drosophila miRNA pathway. RNA.

[31] Huang B, Jones SA, Brandenburg B, Zhuang X (2008). Whole-cell 3D STORM reveals interactions between cellular structures with nanometer-scale resolution. Nat. Methods.

[32] Zearfoss NR, Ryder SP (2012). End-labeling oligonucleotides with chemical tags after synthesis. Methods Mol. Biol..

[33] Tseng Q (2012). Spatial organization of the extracellular matrix regulates cell-cell junction positioning. Proc. Natl Acad. Sci. USA.

[34] Bronson JE, Fei J, Hofman JM, Gonzalez RL, Wiggins CH (2009). Learning rates and states from biophysical time series: a Bayesian approach to model selection and single-molecule FRET data. Biophys. J..

