# Supplementary material for: Single-molecule analysis of processive double-stranded RNA cleavage by Drosophila Dicer-2
Source: Nat Commun. 2021 Jul 13;12:4268. doi: 10.1038/s41467-021-24555-1 (PMC8277814; doi:10.1038/s41467-021-24555-1)
Supplement: Supplementary file 1 — Supplementary Figures 1-6 and Supplementary Table 1 [file 41467_2021_24555_MOESM1_ESM.pdf]

Supplementary Information

Single-molecule analysis of processive double-stranded RNA cleavage by *Drosophila* Dicer-2

Masahiro Naganuma, Hisashi Tadakuma, Yukihide Tomari

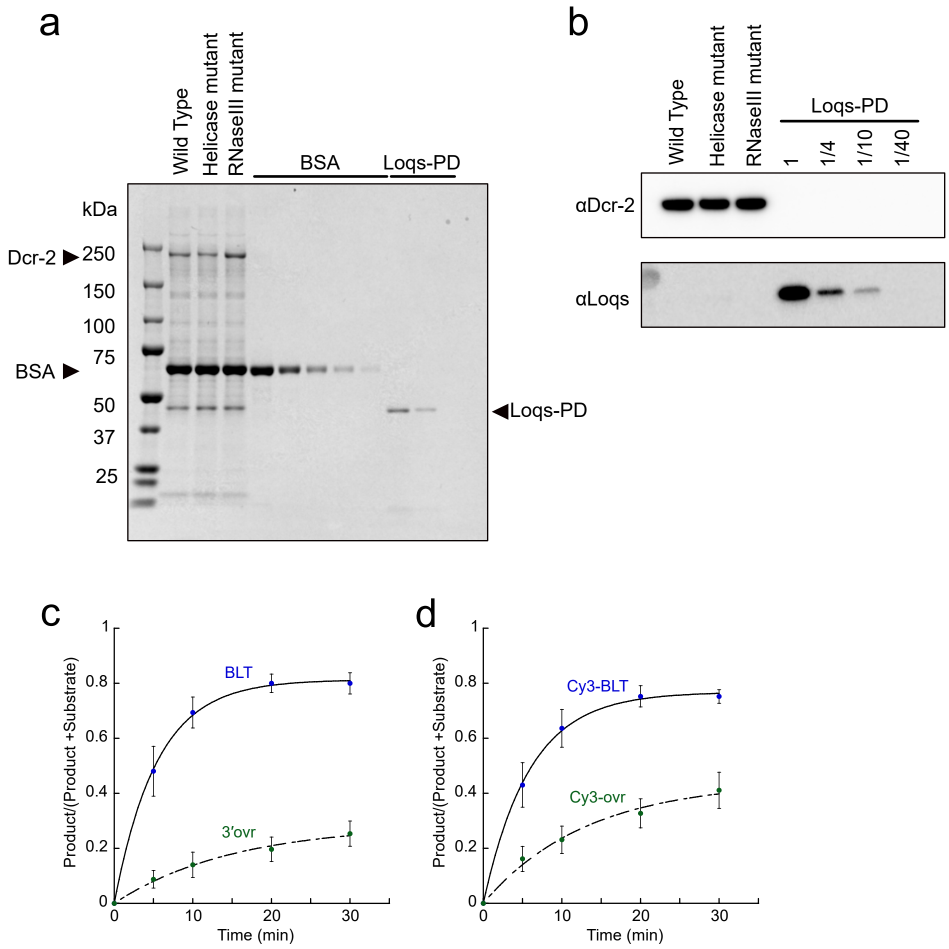

Supplementary Figure 1 Preparation of Dcr-2 and Loqs-PD proteins. a, Coomassie-stained SDS-PAGE of Dcr-2 and Loqs-PD. *n* = 1. Source data are provided as a Source Data file. **b**, No apparent contamination of Loqs-PD was detected in the Dcr-2 preparations by the western blot. The molar ratios of Loqs-PD relative to Dcr-2 are shown above lanes of Loqs-PD. *n* = 2 independent experiments. Source data are provided as a Source Data file. **c, d**, Quantification of the dicing assay in Fig. 1e for non-labeled dsRNAs (**c**) and Cy3-labeled dsRNAs (**d**). Data from three independent trials are presented as mean values ± SD. Source data are provided as a Source Data file.

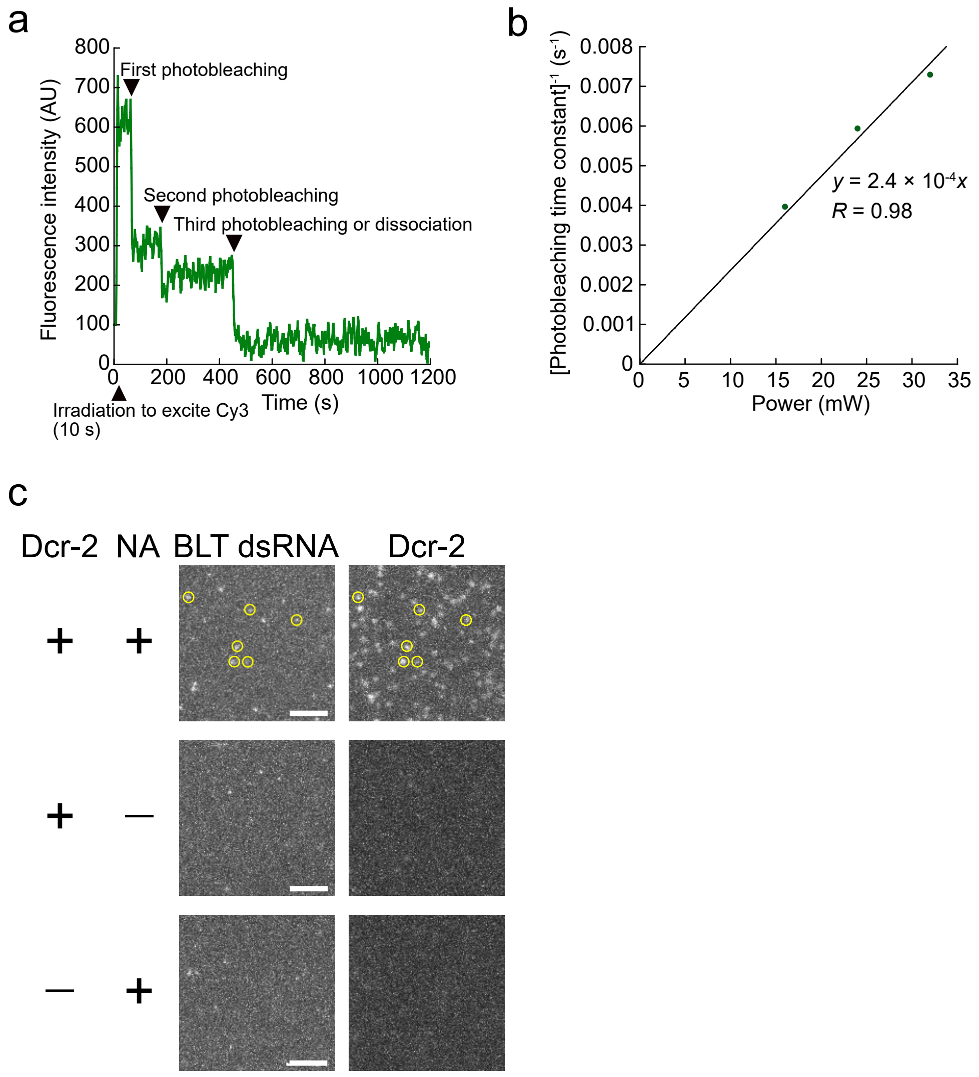

Supplementary Figure 2 Analyses of the RNase III mutant and photobleaching a, Representative trace of photobleaching for 3× Cy3-labeled BLT dsRNAs using the RNase III mutant and ATP-γS, which inhibits dsRNA translocation of the helicase domain. **b,** Photobleaching dependence on power. The photobleaching time constants of Cy3 under the continuous monitoring condition were estimated as 1042 s, which is sufficiently longer than the duration of the whole dicing reaction (~130 s). Source data are provided as a Source Data file. **c,** Representative single-molecule images of surface-tethered Dcr-2 for negative controls (Dcr-2 or NeutrAvidine (NA) was omitted). Scale bar, 4 μm; inset.

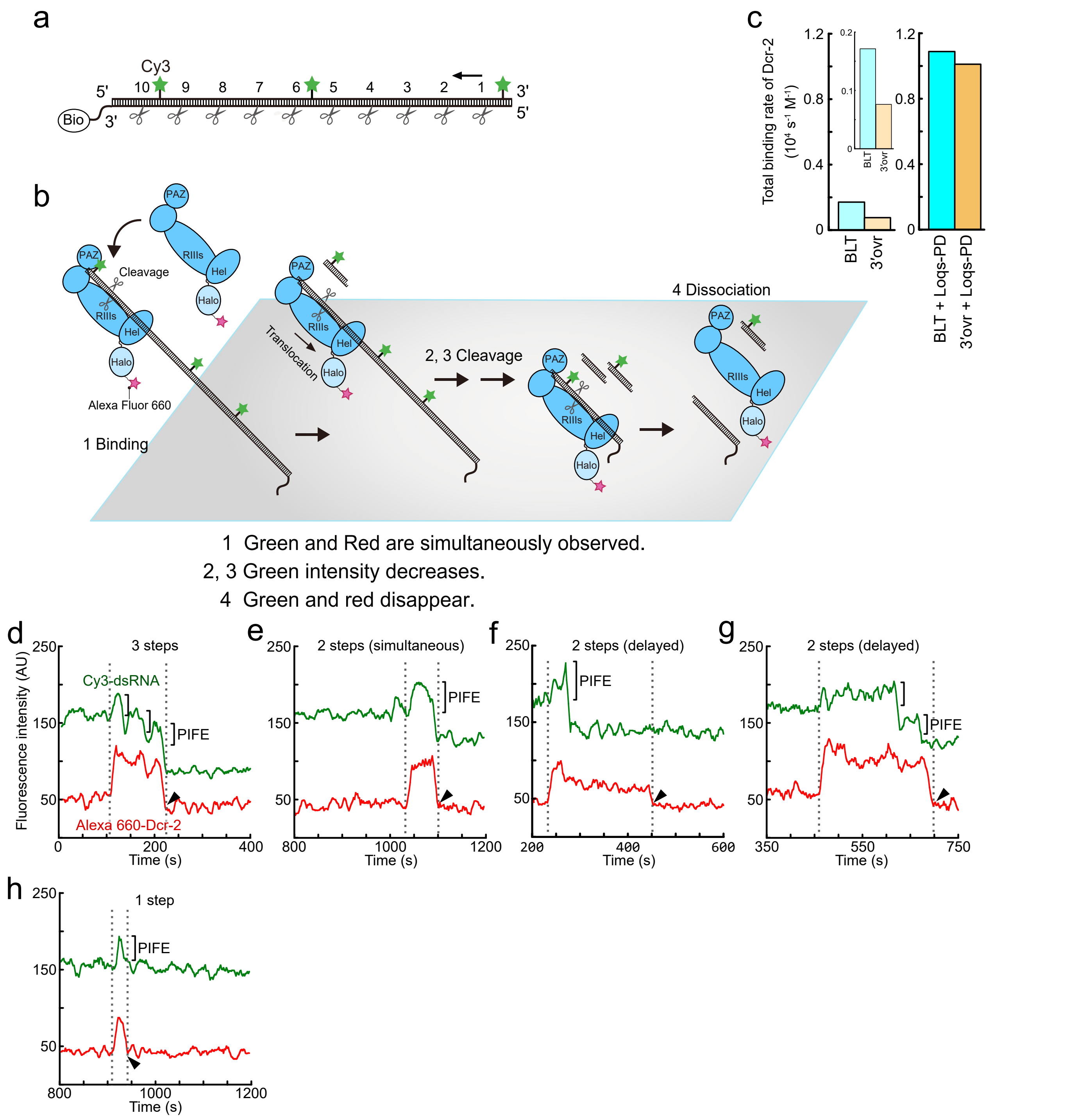

Supplementary Figure 3 Single-molecule analysis of the dicing reaction with the dsRNA tethered on the glass. a, Schematic of the dsRNA substrate labeled with 3× Cy3 at designated positions. "Bio" represents biotin to tether on the glass surface. Scissors represent cleavage sites. **b,** Schematic representation of the dsRNA-anchored single-molecule observation. **c,** Binding rate of Dcr-2, calculated from the total number of binding events (3-steps, 2-steps, and 1-step). Source data are provided as a Source Data file. **d–h,** Representative trace of the "3-steps" (**d**), "2-steps (simultaneous)" (**e**), "2-steps (delayed)" (**f** and **g**) and "1-step" (**h**) events. "2-steps (simultaneous)" is defined as the cleavage events in which Dcr-2 dissociation is accompanied with a single-step cleavage of dsRNA (**e**). "2-steps (delayed)" is defined as the events in which Dcr-2 dissociation occurs after a single-step cleavage of dsRNA as shown (**f** and **g**). PIFE (of the second dye in the dsRNA) may (**g**) or may not (**f**) occur, depending on where Dcr-2 drops off the dsRNA. Black arrowheads indicate the dissociation of Alexa 660-labled Dcr-2.

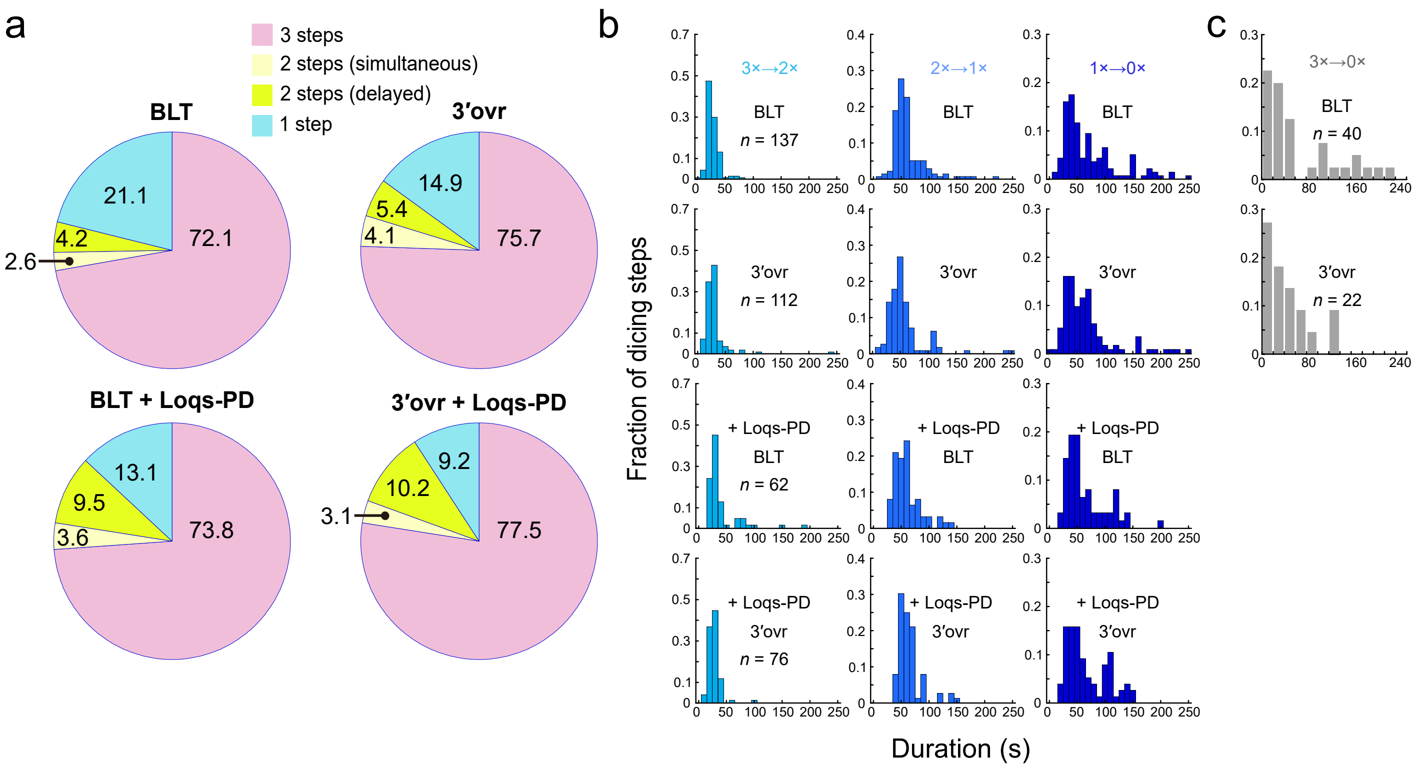

Supplementary Figure 4 Characterization of single-molecule events with the dsRNA tethered on the glass. a, Pie-charts showing the proportions of the "3-steps", "2-steps (simultaneous)", "2-steps (delayed)", and "1-step" events. Source data are provided as a Source Data file. **b,** Dwell time histograms of the 3×→2×, 2×→1×, and 1×→0× steps in the "3-steps" events. Source data are provided as a Source Data file. **c**, Dwell time histograms of the "1-step" events. Please note that the +Loqs-PD data were not plotted, as the event number was very small (*n* = 11 and 9 for BLT and 3′ovr, respectively). Source data are provided as a Source Data file.

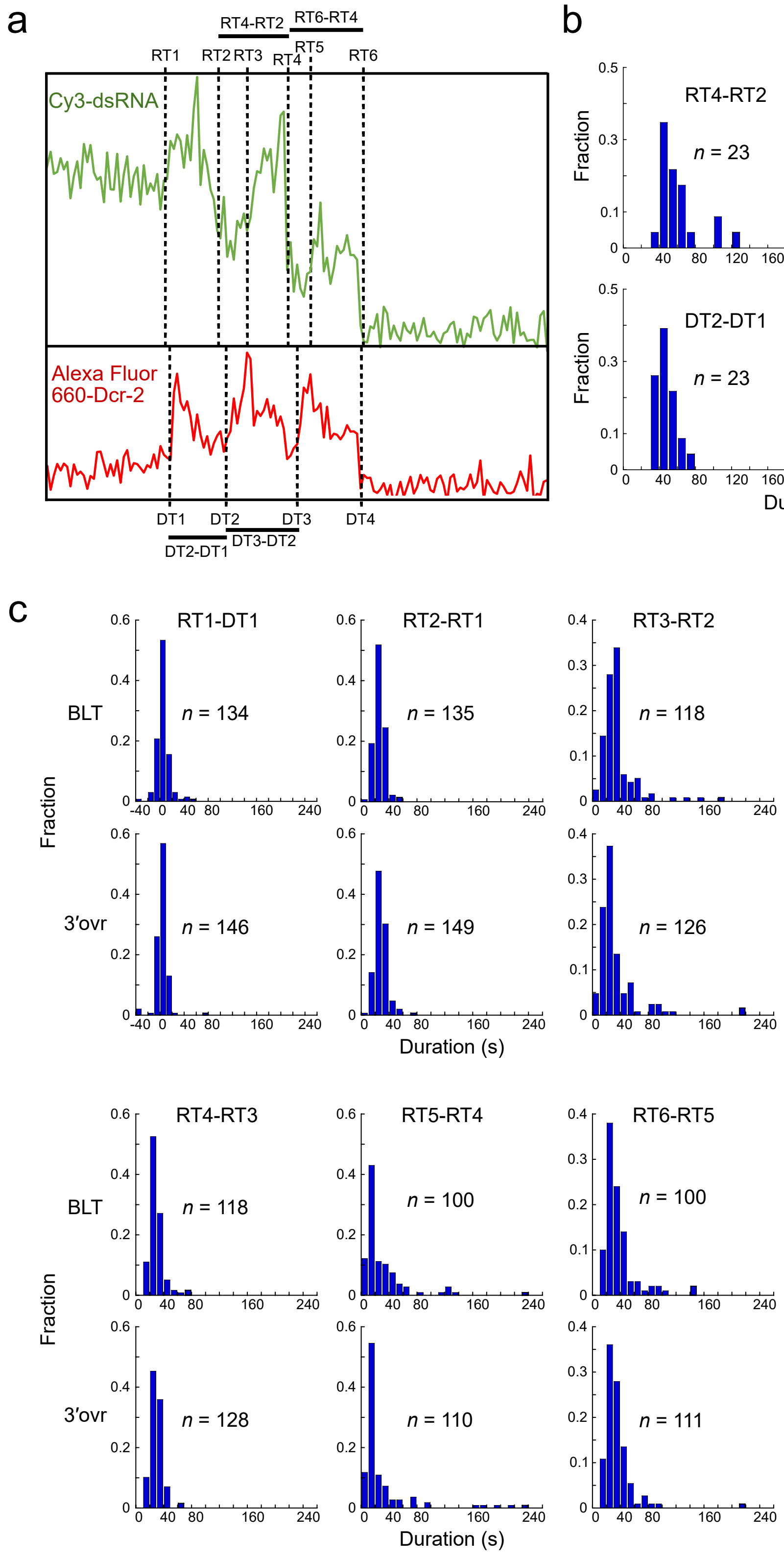

Supplementary Figure 5 PIFE and FRET analyses for dsRNA-anchored single-molecule observation. a, Representative trace of the “3-steps” event. The definitions for the PIFE analysis (RT1-6 from Cy3-dsRNA channel) and FRET analysis (DT1-4 from Alexa 660-Dcr-2 channel) are indicated. **b**, RT4-RT2 and RT6-RT4 should represent the time for 5- and 4-times cleavage, respectively. DT2-DT1 and DT3-DT2 should represent the time required for Dcr-2 to translocate between the first and second Cy3-dye, and the second and third Cy3-dye, respectively. In theory, RT4-RT2 is equal to DT2-DT1 and RT6-RT4 is equal to DT3-DT4, which is supported by the actual data. Source data are provided as a Source Data file. **c**, Histograms of the PIFE duration. Source data are provided as a Source Data file.

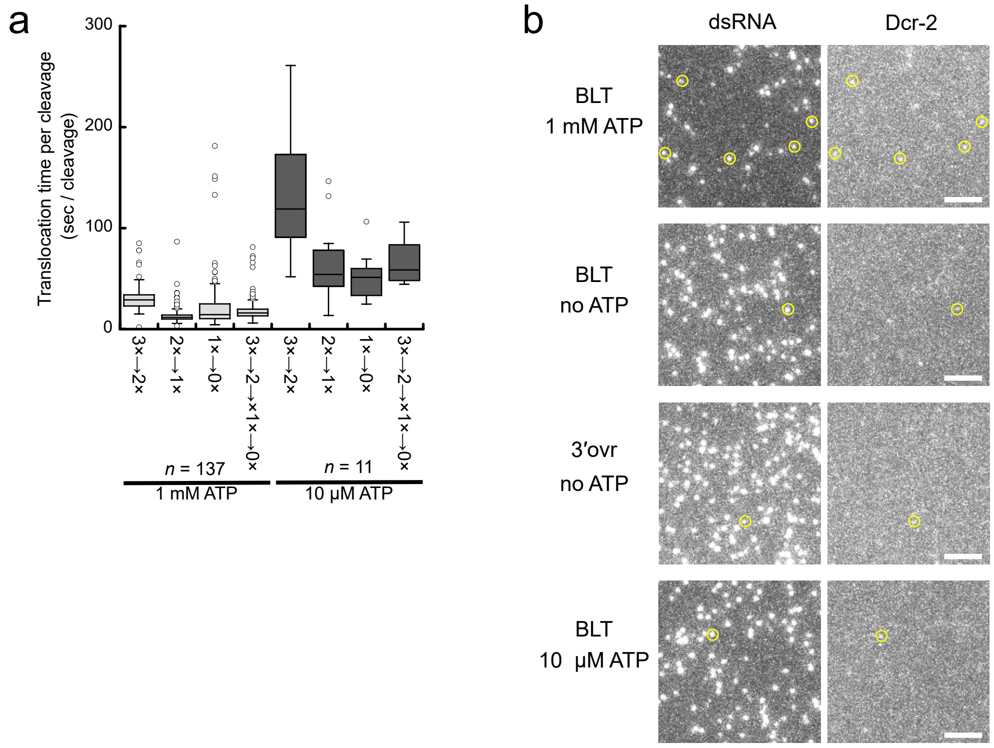

Supplementary Figure 6 Effect of ATP on the translocation speed and binding frequency in the dsRNA-anchored single-molecule experiment. a, Translocation time (sec/cleavage) of Dcr-2 with 1 mM or 10 μM ATP. Box-plot indicates the 1st and 3rd quartiles (upper and lower bounds), 2nd quartile (center), 1.5 × interquartile range (whiskers) and outliers (circles). Source data are provided as a Source Data file. **b**, Representative single-molecule images of surface-tethered dsRNA. Types of dsRNA (BLT or 3′ovr) and ATP concentrations are indicated. Co-localized spots were indicated by yellow circles. BLT dsRNAs were frequently co-localized with wild-type Dcr-2 in the 1 mM ATP condition (*n* = 10), but rarely in the 10 μM (*n* = 2) and no ATP conditions (*n* = 1 for BLT and *n* = 1 for 3′ovr). Scale bar, 4 μm; inset.

Primer Name	Purpose	Sequence
Dcr-2 G31R Fw	QuikChange	CCCACACGATCTGGGAAAACGTTCGTG
Dcr-2 G31R Rv	QuikChange	CCCAGATCGTGTGGGCAGGTAGACAAT
Dcr-2_D1217A_Fw	QuikChange	CTGGGCGCTTCCTTCTTAAAACTTAGT
Dcr-2_D1217A_Rv	QuikChange	GAAGGAAGCGCCCAGAATCTCAAGGCG
Dcr-2_D1476A_Fw	QuikChange	ATTGGCGCTGCCATACTCGACTTTCTA
Dcr-2_D1476A_Rw	QuikChange	TATGGCAGCGCCAATAAATTCCAGCTC
FwFw	Amplification of templete	GTACTTAATACGACTCACTATAGGGCCTCAAGACAGCGAG
FwRv	Amplification of templete	G(2′-*O*-Me-G)GCTTGACGTTGCCGG
RvFw_BLT	Amplification of templete	GTACTTAATACGACTCACTATAGGGCTTGACGTTGCCGGT
RvRv_BLT	Amplification of templete	G(2′-*O*-Me-G)GCCTCAAGACAGCGAG
RvFw_3ovr	Amplification of templete	GTACTTAATACGACTCACTATAGCTTGACGTTGCCGGTGG
RvRv_3ovr	Amplification of templete	T(2′-*O*-Me-T)GGGCCTCAAGACAGCGAG
FwFw_dsRNA-fix	Amplification of templete	GTACTTAATACGACTCACTATAGGGCCACAAGACAGCGAG
RvRv_BLT_dsRNA-fix	Amplification of templete	G(2′-*O*-Me-G)GCCACAAGACAGCGAG
RvRv_3ovr_dsRNA-fix	Amplification of templete	T(2′-*O*-Me-T)GGGCCACAAGACAGCGAG

Supplementary Table 1. Primers used in this study.
