# Supplementary material for: Single-molecule analysis of processive double-stranded RNA cleavage by Drosophila Dicer-2
Source: Nat Commun. 2021 Jul 13;12:4268. doi: 10.1038/s41467-021-24555-1 (PMC8277814; doi:10.1038/s41467-021-24555-1)
Supplement: Supplementary file 2 — Reporting Summary [file 41467_2021_24555_MOESM2_ESM.pdf]

nature research

Corresponding author(s): Yukihide Tomari

Last updated by author(s): Jun 12, 2021

Reporting Summary

Nature Research wishes to improve the reproducibility of the work that we publish. This form provides structure for consistency and transparency in reporting. For further information on Nature Research policies, see our Editorial Policies and the Editorial Policy Checklist.

Statistics

For all statistical analyses, confirm that the following items are present in the figure legend, table legend, main text, or Methods section.

n/a	Confirmed	
☐	☒	The exact sample size (n) for each experimental group/condition, given as a discrete number and unit of measurement
☐	☒	A statement on whether measurements were taken from distinct samples or whether the same sample was measured repeatedly
☒	☐	The statistical test(s) used AND whether they are one- or two-sided<br>*Only common tests should be described solely by name; describe more complex techniques in the Methods section.*
☒	☐	A description of all covariates tested
☒	☐	A description of any assumptions or corrections, such as tests of normality and adjustment for multiple comparisons
☐	☒	A full description of the statistical parameters including central tendency (e.g. means) or other basic estimates (e.g. regression coefficient) AND variation (e.g. standard deviation) or associated estimates of uncertainty (e.g. confidence intervals)
☒	☐	For null hypothesis testing, the test statistic (e.g. F, t, r) with confidence intervals, effect sizes, degrees of freedom and P value noted<br>*Give P values as exact values whenever suitable.*
☐	☒	For Bayesian analysis, information on the choice of priors and Markov chain Monte Carlo settings
☒	☐	For hierarchical and complex designs, identification of the appropriate level for tests and full reporting of outcomes
☐	☐	Estimates of effect sizes (e.g. Cohen's d, Pearson's r), indicating how they were calculated

Our web collection on statistics for biologists contains articles on many of the points above.

Software and code

Policy information about availability of computer code

Data collection	Andor SOLIS 4.28.30026
Data analysis	MATLAB R2019a, vbFRET version nov12 (an open source MATLAB package, Bronson et al. doi: 10.1016/j.bpj.2009.09.031), ImageJ 1.47, Excel 2016, KaleidaGraph v3.6

For manuscripts utilizing custom algorithms or software that are central to the research but not yet described in published literature, software must be made available to editors and reviewers. We strongly encourage code deposition in a community repository (e.g. GitHub). See the Nature Research guidelines for submitting code & software for further information.

Data

Policy information about availability of data

All manuscripts must include a data availability statement. This statement should provide the following information, where applicable:

- Accession codes, unique identifiers, or web links for publicly available datasets
- A list of figures that have associated raw data
- A description of any restrictions on data availability

The source data underlying Figs. 1b–e, 2j, 3a, c, d and Supplementary Figs. 1a–d, 2b, 3c 4a–c, 5b, c and 6a are provided as a Source Data file.

April 2020

Field-specific reporting

Please select the one below that is the best fit for your research. If you are not sure, read the appropriate sections before making your selection.

☒ Life sciences ☐ Behavioural & social sciences ☐ Ecological, evolutionary & environmental sciences

For a reference copy of the document with all sections, see nature.com/documents/nr-reporting-summary-flat.pdf

Life sciences study design

All studies must disclose on these points even when the disclosure is negative.

Sample size	No statistical methods were used to predetermine the sample size, because as much single-molecule data as possible were collected to ensure sufficient sampling for subsequent analyses.
Data exclusions	For single molecule analysis, some of the data were excluded due to poor imaging quality that is insufficient for the further analysis. For example, traces in which the Cy3 intensity was less than 3x were excluded.
Replication	Bulk dicing assays were independently performed 1–3 times, depending on the nature of the experiments. Single-molecule observations were replicated 4–20 times. The numbers of replications are described in Figure Legends.
Randomization	Randomization was not performed in this study, because there is no statistical method that requires randomization of samples.
Blinding	Blinding was not required in this study, because sample preparation, data collection and image analysis were performed using the same conditions for all the samples regardless of their identity.

Reporting for specific materials, systems and methods

We require information from authors about some types of materials, experimental systems and methods used in many studies. Here, indicate whether each material, system or method listed is relevant to your study. If you are not sure if a list item applies to your research, read the appropriate section before selecting a response.

Materials & experimental systems

n/a	Involved in the study	
☐	☒	Antibodies
☐	☒	Eukaryotic cell lines
☒	☐	Palaeontology and archaeology
☒	☐	Animals and other organisms
☒	☐	Human research participants
☒	☐	Clinical data
☒	☐	Dual use research of concern

Methods

n/a	Involved in the study	
☒	☐	ChIP-seq
☒	☐	Flow cytometry
☒	☐	MRI-based neuroimaging

Antibodies

Antibodies used	[Primay antibodies] Anti-Dcr-2 and anti-Loqs antibodies were originally produced and kindly provided by Mikiko Siomi (The University of Tokyo, Japan) and Haruhiko Siomi (Keio University, Japan).<br>[Secondary antibody] Anti-IgG (H+L chain) (Mouse) pAb-HRP (MBL) was purchased.
Validation	[Primay antibodies] Anti-Dcr-2 and Loqs antibodies were validated in a previous study (Miyoshi et al. doi: 10.1261/rna.1541209).<br>[Secondary antibody] Anti-IgG (H+L chain) (Mouse) pAb-HRP (MBL) (https://ruo.mbl.co.jp/bio/dtl/dtlfiles/330_v5.pdf)

Eukaryotic cell lines

Policy information about cell lines

Cell line source(s)	S2 cells were purchased from Invitrogen.
Authentication	S2 cells have not been authenticated.
Mycoplasma contamination	Not tested.
Commonly misidentified lines<br>(See ICLAC register)	No commonly misidentified cell lines were used.
